# A national measles outbreak in Ireland linked to a single imported case, April to September, 2016

**DOI:** 10.2807/1560-7917.ES.2018.23.31.1700655

**Published:** 2018-08-02

**Authors:** Peter Barrett, Suzanne Cotter, Fiona Ryan, Jeff Connell, Anthony Cronin, Mary Ward, Rose Fitzgerald, Catherine Lynch, Tony Margiotta, on behalf of the Outbreak Control Team

**Affiliations:** 1 Department of Public Health HSE South, St. Finbarr’s Hospital, Cork, Ireland; 2 Health Protection Surveillance Centre, Dublin, Ireland; 3 National Virus Reference Laboratory, University College Dublin, Dublin, Ireland; 4 Department of Public Health HSE East, Dr. Steevens’ Hospital, Dublin, Ireland; 5 Department of Public Health HSE Mid-West, Limerick, Ireland; 6 Department of Public Health HSE South-East, Kilkenny, Ireland; 7 Department of Public Health, HSE North-East, Navan, Co. Meath, Ireland; 8 The members of the Outbreak Control Team are acknowledged at the end of the article

**Keywords:** measles, measles-mumps-rubella (MMR) vaccine, outbreaks, vaccine-preventable diseases

## Abstract

Endemic measles transmission was interrupted for the first time in Ireland in 2015. In May 2016, a case of measles was confirmed in an adult who had travelled from Hungary to Ireland (index case). Cases subsequently arose in five of the eight public health regions around the country. There were 40 confirmed cases in Ireland between April and September 2016. All sequenced cases were genotype B3. Vaccination status was known for 34 cases, of whom 31 were unvaccinated. Median age was 8 years (range: 3 months to 40 years). Ten cases were nosocomial, and three cases were infected on separate international flights. One linked case occurred in a resident of Slovenia. Nineteen cases were hospitalised; median duration of hospitalisation was 5 days (range: 2–8 days). The primary case was a child who travelled from Romania to Ireland via Budapest, and infected the index adult case on the same flight. This was the first reported outbreak of measles genotype B3 in Ireland. This outbreak demonstrated that Ireland remains at risk of measles outbreaks due to persistent suboptimal vaccination rates.

## Background

In Ireland, the incidence of measles has decreased since the introduction of the measles vaccine in 1985. The incidence fell from 84 cases per million in 2004 to 7 cases per million in 2014 [1,2]. In 2015, the World Health Organization (WHO) elimination target (< 1 case per million) was met for the first time in Ireland and endemic transmission was interrupted when two confirmed cases of measles were reported that year [3,4]. In the first quarter of 2016 there was one imported case of measles notified [5].

It is increasingly recognised that one of the greatest risks posed to countries approaching measles elimination is importation of infectious cases. On 9 May 2016, a confirmed case of measles was reported to the regional Department of Public Health in Dublin, in an adult who had returned from Hungary 3 weeks previously. On return to Ireland, this case travelled extensively in the south-west of the country while symptomatic with rash and fever. In the 2 weeks following this notification, 10 additional cases of measles were notified. A national outbreak was declared, and continued until September 2016.

The initial steps involved in the investigation of this outbreak have been described previously in a rapid communication [5]. The purpose of this final outbreak report is to describe in greater detail all cases in this outbreak, including 13 cases which arose subsequent to the rapid communication. The sequence of events which occurred in the outbreak is described, and factors which facilitated onward spread of disease are identified. Furthermore, the strengths and weaknesses of ongoing measles control efforts in Ireland are considered, based on the lessons learnt from an evaluation of this outbreak.

## Methods

### Case definition and laboratory diagnosis

Cases were defined as possible, probable or confirmed, depending on clinical, epidemiological and laboratory criteria [6]. Possible cases were those who met the clinical criteria of fever, maculopapular rash, and at least one of cough, coryza and conjunctivitis. Probable cases were those who met clinical criteria and had an epidemiological link to a case by human-to-human transmission. Confirmed cases were those who met clinical criteria and had a confirmed laboratory diagnosis of measles.

A variety of samples were used to confirm or rule out measles; primarily oral fluid samples collected using the OraCol collection device (Malvern Medical Developments, Worcester, United Kingdom (UK)), serum or throat swabs. The type of sample obtained from patients was determined by the time between onset of rash and time of sample. The National Virus Reference Laboratory (NVRL) in Dublin performed all diagnostic investigation for suspect cases. Oral fluid specimens collected within 7 days of rash onset were investigated for measles RNA using RT-PCR. Oral fluid samples collected 5 days or more after rash onset, and serum specimens collected more than 3 days after rash onset, were tested for measles IgM using a measles IgM capture enzyme immunoassay (Microimmune, Hounslow, Middlesex, UK). When measles RNA was not detected from possible cases, oral fluid was tested for HHV-6 DNA, Enterovirus RNA, Parvovirus DNA and Parechovirus RNA (in children under 3 years of age).

Measles genotyping was performed by analysing the sequence of the N-450 of the virus. All cases sequenced and uploaded into WHO Measles Nucleotide Surveillance (MeaNS) were checked to identify identical sequences elsewhere, and to determine if links existed to other cases [7]. Genotyping was undertaken for the primary case, and for any cases whose epidemiological links to the outbreak were uncertain.

### Contact tracing and post-exposure prophylaxis

Extensive contact tracing was undertaken for all probable and confirmed cases. Contact tracing was also undertaken for possible cases with a strong index of clinical suspicion (i.e. those with non-localised, diffuse maculopapular rash). Eligibility for prophylactic measles-mumps-rubella (MMR) vaccination and human normal immunoglobulin (HNIG) was assessed for all contacts. Those who were not vaccinated appropriately for their age (i.e. first dose MMR at 12 months, second dose at age 4–5 years) were eligible for prophylactic MMR vaccination if they could receive the vaccine within 72 hours of first contact with an infectious case of measles. HNIG was considered for immunocompromised contacts, unvaccinated pregnant women, and selected infants under 12 months of age if they had had contact with an infectious case in the previous 6 days [8].

### Data collection and analysis

Regional Departments of Public Health collected enhanced surveillance information on all notified cases, and this information was uploaded to the national Computerised Infectious Disease Reporting system (CIDR). Descriptive analyses of surveillance data were performed using Excel. Results are reported as frequencies, and median values with interquartile ranges (IQR) are reported for continuous variables.

After the outbreak was closed an evaluation of overall outbreak management was undertaken. A short questionnaire was sent to all members of the Outbreak Control Team (OCT) in each region. This evaluation has led to the documentation of specific challenges that need to be addressed to reduce the risk of future measles outbreaks.

## Results

### Descriptive epidemiology

Between 9 May and 2 September 2016 there were 40 laboratory-confirmed cases of measles linked to the outbreak in Ireland. One further confirmed case in a resident of Slovenia, exposed on a flight, was linked to this outbreak. This case was reported by Slovenian national public health authorities, but is not included in the final results of the outbreak in Ireland.

**Figure 1:**
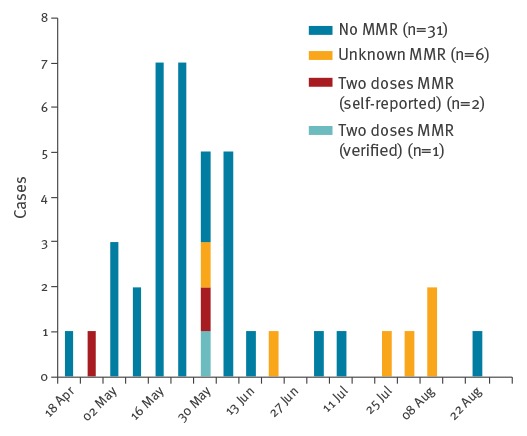
Epidemic curve of confirmed cases by vaccination status in national measles outbreak, Ireland, April–September, 2016 (n = 40)

The demographic characteristics of affected cases are summarised in the Table. Where vaccination status was known, most (31/34) were unvaccinated. The median age for all cases was 8 years (IQR: 10 months–31 years). Eight cases were aged under 12 months. Among those whose MMR vaccination status was unknown, median age was 32 years (range: 24–40 years). The epidemic curve for the outbreak is shown by MMR vaccination status in Figure 1.

**Table:** Demographic details of confirmed cases in national measles outbreak, Ireland, 2016 (n = 40)

Variable	Total (n = 40)
**Age group (years)**	
< 1	8
1–4	5
5–9	8
10–14	4
15–19	6
20–29	4
≥ 30	5
**Sex**	
Male	21
Female	19
**Country of birth**	
Ireland	25
Outside of Ireland	11
Unknown	4
**MMR vaccination status**	
Two doses: verified	1
Two doses: self-reported	2
One dose	0
None^a^	31
Unknown	6

MMR: measles-mumps-rubella.

**^a^** Eight of these cases were < 1 year of age, and under the recommended age for routine MMR vaccination.

MMR: measles-mumps-rubella.

**Figure 2:**
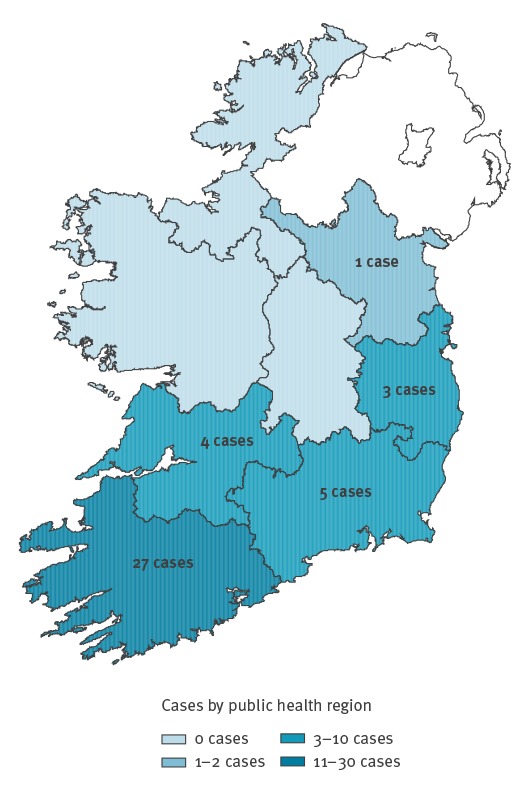
Map of confirmed cases by public health regions in national measles outbreak, Ireland, April–September, 2016 (n = 40)

Cases occurred in five of the eight public health regions of Ireland. The greatest number of cases occurred in the south-west of the country (n = 27), particularly County Kerry (Figure 2). Thirteen cases resident in the south-west of Ireland were from the Roma community.

**Figure 3:**
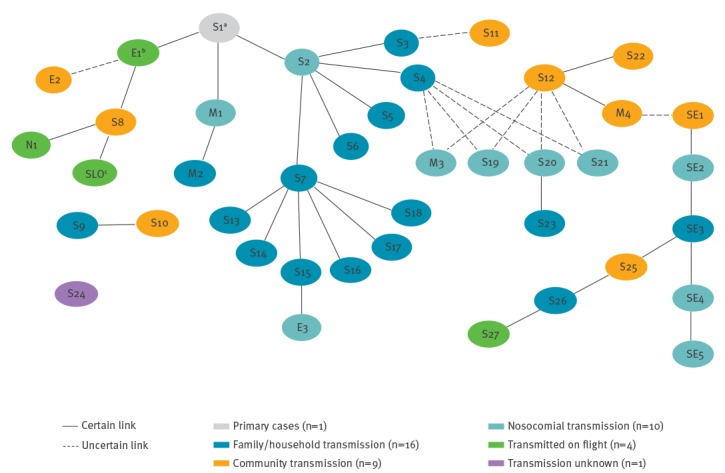
Epidemiological links between confirmed cases in national measles outbreak, Ireland, April–September, 2016 (n = 40)

Oral fluid specimens were obtained for testing from all 40 confirmed cases. Serological investigation for measles IgM was also undertaken in 16 cases. The median duration from date of rash onset to date of notification on CIDR was 7 days (IQR: 3–8 days). The epidemiological links between all confirmed cases in the outbreak are shown in Figure 3.

Geographic location: S: South; E: East; SE: South-East; M: Mid-West; N: North-East.

^a^ Primary case.

^b^ Index case.

^c^ Slovenian case.

One case in a Slovenian resident was reported by Slovenian national public health authorities, and is not included in analyses of the 40 outbreak cases in Ireland.

Most confirmed cases (26/40) were assessed in hospital. Nineteen cases were hospitalised as a result of measles, of whom 12 were male. The median duration of hospitalisation was 5 days (IQR: 3–6 days). The median age of hospitalised cases was 10 years (IQR: 10 months–28 years). One case developed pneumonia, and one case developed acute respiratory distress. There were no reported cases of seizures, meningitis or encephalitis, and there were no deaths.

In addition to confirmed cases, there were 114 possible cases notified between 1 May and 30 September 2016 which were subsequently denotified following investigation and negative results for measles.

### Outbreak investigation

#### Identification of the primary case

After Case E1 (index case) was notified on 9 May, a public health investigation was initiated. Case E1 had travelled from Budapest to Dublin 3 weeks previously and became symptomatic with fever and rash upon return. Case E1 travelled extensively in Ireland while infectious.

On 13 May, Case M1 was notified. Case M1 had been a hospital inpatient in Kerry for an unrelated illness the previous month.

Extensive follow up of contacts and case finding identified Case S1. This child had travelled from Romania to Ireland via Hungary on the same flight as Case E1. Case S1 had been unwell with a fever and rash on the flight to Ireland, and was then hospitalised in Kerry for an unspecified febrile rash illness. Case S1 was not investigated for measles on admission to hospital, and was not isolated. When Case S1 was suspected as the primary case, the NVRL retrieved a throat swab taken during admission. This had been originally investigated for influenza, but measles RNA was detected in this sample when it was tested retrospectively.

The Health Protection Surveillance Centre (HPSC) liaised with Romanian authorities regarding the areas visited by Case S1. It was confirmed that one of the villages in western Romania which Case S1 had visited had a measles outbreak at that time. Case S1 had been in contact with a child with fever and rash while there, and was thus confirmed as the primary case in the outbreak in Ireland.

#### Identification of transmission chains

As shown in Figure 3, family/household transmission was the most frequent transmission pathway and resulted in 16 cases. Nine confirmed cases were most likely to have been acquired in community settings. For example, Case S8 had visited the same village as Case E1 when Case E1 was infectious (i.e. shortly before/during onset of rash). Ten confirmed cases were nosocomial infections, involving two hospitals: six hospital patients, two healthcare workers and two hospital visitors.

Four confirmed cases, linked to the outbreak, were known to have acquired the infection on three separate international flights into Ireland (i.e. index case and three others). One of these cases was a resident of Slovenia, who was not otherwise counted in the final results. On two of the respective flights, a previously exposed Irish resident travelled out of Ireland during the incubation period and returned to Ireland while infectious, thereby exposing other passengers during transit. In addition, another confirmed case (Case E3) took two flights during their infectious period but no known onward transmission occurred; 250 contacts were followed up but no further cases were identified.

For one of the outbreak cases (S24), no epidemiological links could be established. No genotyping information was available for this case, thus we cannot rule out the possibility that this was a false-positive result.

#### Molecular surveillance

Measles sequencing was performed for the sample available for Case S1, and genotype B3 was identified. In total, sequences from 33 cases were uploaded into MeaNS from the outbreak; these cases were all genotype B3 and 100% identical.

### Outbreak control measures

#### Case follow-up and contact tracing

Confirmed and suspected cases were advised to avoid contact with others, particularly vulnerable groups, until 5 days after rash onset, and to telephone healthcare workers in advance of seeking care. Contacts of suspected/confirmed cases were sent letters to alert them of the possibility of symptoms, and to outline necessary precautions to reduce their risk of acquiring measles. Letters emphasised the need for age-appropriate MMR vaccination among all contacts.

MMR vaccines were not generally administered to contacts earlier than planned per the routine schedule, unless they received an MMR dose as post-exposure prophylaxis within 72 hours. Contacts born before 1978 were not advised to get MMR vaccination due to the high probability that they had natural immunity as a result of childhood infection [9].

The contacts eligible for prophylactic MMR were not enumerated. However, these numbers were small due to delayed (> 72 hours) notification of cases. Larger numbers of contacts received age-appropriate catch-up vaccination outside of this 72-hour window. No contacts met eligibility criteria for prophylactic HNIG.

#### Communication

There was proactive communication with parents and guardians of children who may have had contact with infectious cases through school, crèche or other educational settings. Information was also sent to patients who may have been exposed to infectious measles cases in waiting rooms, emergency rooms or on hospital wards.

Alerts were emailed to local clinical networks to inform them of the outbreak, and to inform clinicians of the process for measles testing and notification. Departments of Public Health also liaised with local hospital occupational health colleagues to inform them of the outbreak. Hospital management were encouraged to exclude unvaccinated healthcare workers from clinical work where possible.

When Case E1 was notified, a national press statement was released to raise public awareness of measles, given the individual’s extensive travel. There were seven further national press releases to update the public of the ongoing risk of measles. Radio interviews were conducted at a local and national level to respond to queries. Updates were also sent out via social media.

Communication with airports and airlines was required as a control measure since infectious measles cases had travelled on five different flights. Airport management teams were advised to inform their staff of the possibility of exposure to measles in the terminal building. In addition, HPSC liaised with airlines directly to inform them of the risk to exposed passengers and crew. All airlines which carried an infectious case agreed to send out text messages or email alerts to passengers and crew to inform them of their possible exposure, and advised on actions to take in the event of becoming symptomatic.

International communication to other European Union (EU) countries and the WHO Regional Office for Europe was made using the EU Early Warning and Response System (EWRS) on 2 June 2016.

#### Health promotion

Regional Departments of Public Health and the HPSC collaborated with community workers to produce information leaflets about measles for the general public and for specific subgroups. In the first month of the outbreak, a disproportionately large number of cases were reported from the Roma community, particularly in the south-west of Ireland. Consequently, leaflets were translated into the four dominant languages of the Roma communities in the south-west region: Czech, Polish, Romanian and Slovakian.

Posters were also developed by HPSC to raise awareness among healthcare workers and patients of the ongoing risk of measles transmission. These posters were distributed to hospitals and general practitioner (GP) surgeries.

## Discussion

This was a complex, protracted outbreak involving 40 confirmed cases of measles which demonstrated the ongoing vulnerability among the Irish population to measles infection. The majority of cases were unvaccinated. Children under 5 years old were disproportionately affected, and the youngest case was 3 months old. Only one case arose in an adult born before 1978. Extensive contact tracing allowed the primary case to be identified. The primary case had visited a village in western Romania where an ongoing outbreak of measles genotype B3 was confirmed [10].

There were multiple venues and settings in which transmission occurred. This was the first reported measles outbreak in Ireland involving confirmed cases on international flights, and with in-flight transmission to other passengers. Ten cases arose due to nosocomial transmission, and two healthcare workers were infected. Almost half of the cases (19/40) were hospitalised in this outbreak; a higher proportion than in previous Irish outbreaks [11-14]. The median duration of hospitalisation was also longer than previously reported. However, there were fewer recorded clinical complications than documented in previous Irish outbreaks.

Currently, the 95% MMR immunisation target among Irish children remains unmet. Uptake of one MMR dose among 4–5-year-olds in Ireland is 91% [15]. Persistent immunity gaps among recent birth cohorts continue to impede efforts to eliminate measles in Ireland, and there is an ongoing risk of future outbreaks following importation. This was the first time an outbreak of measles genotype B3 was recorded in Ireland. Outbreaks of measles genotype B3 have occurred in Denmark [16], the UK [17] and Italy [18,19] in recent years. Subsequent to this outbreak, cases of this genotype have occurred elsewhere throughout Europe [20-22].

In this outbreak the Roma community was disproportionately affected. In Ireland there is no specific, systematic enumeration of MMR vaccination uptake among ethnic minority groups and therefore the true extent of immunity gaps within Roma in Ireland is unknown. In 2014, an audit of MMR uptake among Roma children in the Dublin region identified that 42% of children eligible for MMR vaccination had documentation of receiving at least one dose of MMR (data not shown). The European Centre for Disease Prevention and Control (ECDC) have highlighted the critical role of Roma health mediators in health promotion efforts and the potential value of using role models or celebrities in community-based information campaigns [23]. Such recommendations should be progressed in order to promote vaccination among these groups.

### Positive aspects of outbreak control

Schools, crèches and primary care centres were cooperative with recommended control measures throughout the outbreak. OCT members perceived that media coverage of the outbreak at the national level was balanced and constructive. The EWRS enabled Slovenian authorities to recognise a linked case in their country, and also facilitated rapid communication when tracing the origin of the outbreak with Romanian colleagues.

Contact tracing efforts helped to prevent onward spread of measles, and allowed epidemiological links between the majority of confirmed cases to be identified. Post-exposure vaccination was administered to susceptible contacts within 72 hours where possible. Catch-up vaccinations were also arranged, and age-appropriate vaccination was promoted for all. The letters sent to contacts helped with more rapid detection, earlier notification, and appropriate isolation of some cases.

Some cases of measles were recognised and reported as a result of text message alerts sent from airlines. For example, the parents of Case S27 reported that they had followed isolation precautions received via text message from their airline when their child had become symptomatic with measles. Case S27’s GP and hospital were warned in advance, thus limiting nosocomial transmission. Case N1, also exposed on a flight, was able to use the information from the text alert to inform their GP that their symptoms might be measles, and public health authorities were alerted immediately.

### Challenges

The requirement for identification and isolation of contacts was not appreciated by all adult contacts, who may have considered measles as a normal childhood illness. Some clinicians and nurses also appeared to underestimate the need for strict isolation and control precautions when dealing with a possible case of measles. Delayed implementation of these measures likely facilitated nosocomial transmission. For example, Cases S4 and S12 were not appropriately isolated in hospital, and there was subsequent nosocomial transmission to four more cases on the same ward. Language barriers also presented a challenge as some affected members of the Roma community spoke little English, and interpreters were not available to assist clinical and public health staff in a timely manner.

There were numerous challenges with delays in diagnosis and laboratory investigation, and these delays likely hampered control efforts. Several cases were not initially recognised as measles during the outbreak possibly due to lack of experience of healthcare workers in dealing with clinical measles cases. Furthermore, diagnostic delay may have occurred due to lack of familiarity among healthcare workers with this diagnosis, as reported in nosocomial measles incidents elsewhere [18,24,25]. When measles was suspected, confirmation of diagnosis was frequently delayed, due to lack of available diagnostic swabs, delayed postage or laboratory processing of samples.

Despite repeated alerts sent via clinical networks, and requests for immediate notification, some notifications continued to be delayed throughout the outbreak. This delay was likely due to a combination of factors among healthcare workers: lack of attention to alerts and communications, inadequate understanding of the role of public health teams in outbreak management, lack of awareness of timeframes for post-exposure prophylaxis, and competing clinical priorities. Some notifications in the outbreak were delayed due to clinicians’ beliefs that notification was only necessary when laboratory confirmation was received.

Vaccine records were not available for all cases in this outbreak, particularly among adults. Of the six cases whose vaccination status was unknown, all were young adults aged 24 to 40 years. Vaccine records were also not available for all healthcare workers. The lack of ready access to immunisation records of hospital staff created difficulties in implementing control measures in this setting. During the course of the outbreak it was identified that most hospital occupational health departments had no standardised mechanism to identify healthcare workers’ vaccination status. Although proof of MMR immunity is recommended as a mandatory requirement, in practice, healthcare workers without evidence of immunity are not prevented from starting work in Irish hospitals. The lack of a single, national database of occupational health records created difficulties in coordinating a national response to nosocomial transmission. Infected healthcare workers may have contributed to nosocomial spread, similar to other measles outbreaks in Europe [26,27].

There was no senior clinician with overall responsibility for infection control in one healthcare facility. This is likely to have contributed to persistent nosocomial transmission within that hospital. The high rate of hospitalisation of cases may have also facilitated nosocomial spread. Inadequate staffing levels precluded precautionary exclusion of unvaccinated healthcare workers from work in some settings.

Isolation facilities were inadequate in some hospitals. One confirmed case was isolated in a single room without an en-suite toilet. This case continued to use shared toilet and shower facilities while infectious and onward nosocomial transmission occurred. Furthermore, relatives visiting infectious cases did not always wear appropriate personal protective equipment because this recommendation was not enforced in all settings.

It is likely that there were other cases of measles which were not reported in this outbreak. For example, Case E2 was most likely infected by an unknown intermediate case, who in turn was most likely infected by Case E1. The problem of under-reporting of measles has been acknowledged elsewhere [28-30].

The full costs associated with the control of this outbreak have not been estimated, but they are likely to be considerable. At least 154 suspect cases had laboratory samples analysed, and hundreds of contacts were investigated. The 19 hospitalised cases in this outbreak accounted for a total of 85 inpatient bed-days in Irish hospitals, two of whom required treatment in the intensive care unit. There were over 50 staff involved in the national OCT at different times. Staff time is likely to comprise the greatest cost component in this outbreak, similar to other outbreaks [31]. The costs of vaccination for measles prevention may be relatively small by comparison with the costs of outbreak management.

## Conclusions

This was the first outbreak of measles genotype B3 reported in Ireland, and it was traced back to a single imported case. The outbreak highlighted the ongoing susceptibility of the Irish population to measles due to persistent immunity gaps among children, young adults, and particularly among vulnerable minority groups. Renewed efforts are needed to build on gains in MMR vaccination coverage which have been achieved over the last decade in Ireland, and to reach the 95% target needed for herd immunity.

This was also the first reported measles outbreak in Ireland involving confirmed cases on international flights coming into Ireland and transmitting to co-passengers. Communication with airline companies as part of control efforts was valuable, and email and text message alerts to exposed passengers and crew helped to limit onward spread of disease. Early cases were not recognised by many clinicians, resulting in delays in diagnoses, notifications and control measures. There is scope to improve measles awareness among healthcare workers, and to ensure that clinical colleagues are appropriately vaccinated with MMR.
